# Dynamic Changes of Intrinsic Brain Activity in Cirrhotic Patients after Transjugular Intrahepatic Portosystemic Shunt: A Resting-State fMRI Study

**DOI:** 10.1371/journal.pone.0046681

**Published:** 2012-10-02

**Authors:** Rongfeng Qi, Long Jiang Zhang, Jianhui Zhong, Shengyong Wu, Zhiqiang Zhang, Yuan Zhong, Ling Ni, Gang Zheng, Qing Jiao, Xingjiang Wu, Xinxin Fan, Yijun Liu, Guangming Lu

**Affiliations:** 1 Department of Medical Imaging, Jinling Hospital, Clinical School of Medical College, Nanjing University, Nanjing, Jiangsu, China; 2 Department of Biomedical Engineering, Zhejiang University, Hangzhou, Zhejiang, China; 3 Medical Imaging Institute of Tianjin, Tianjin, China; 4 Department of General Surgery, Jinling Hospital, Clinical School of Medical College, Nanjing University, Nanjing, Jiangsu, China; 5 Department of Psychiatry, University of Florida McKnight Brain Institute, Gainesville, Florida, United States of America; University of Colorado, United States of America

## Abstract

**Purpose:**

The majority of cirrhotic patients who underwent transjugular intrahepatic portosystemic shunt (TIPS) experienced the first post-TIPS hepatic encephalopathy (HE) episode within the first three months after TIPS insertion. However, so far, little is known about the exact neuro-pathophysiological mechanism of TIPS's effects on brain function. We aimed to investigate the dynamics of brain function alteration of post-TIPS patients using resting-state functional MRI (rs-fMRI).

**Materials and Methods:**

Sixteen cirrhotic patients who were scheduled for TIPS and 16 healthy controls were included in the rs-fMRI scans. Ten patients repeated the MRI study in a median 8-day follow-up interval following TIPS and seven in a median 3-month follow-up. The amplitude of low frequency fluctuation (ALFF), an index reflecting the spontaneous brain activity, was compared between patients before TIPS and healthy controls as well as patients pre- and post- TIPS.

**Results:**

Compared with healthy controls, patients showed decreased ALFF in frontal and parietal regions and increased ALFF in insula. Patients who underwent the median 8-day follow-up fMRI examinations showed decreased ALFF in posterior cingulate cortex (PCC)/precuneus and increased ALFF in anterior cingulate cortex (ACC). Of 10 patients in this group, 9 had moderate to large increase rate of ALFF value (>20%, mean 49.19%) in ACC, while only one patient with the smallest increase rate of ALFF value (<10%) in ACC, who experienced three episodes of overt HE during the 3-month follow-up. In the median 3-month follow up observation, patients displayed persistently decreased ALFF in PCC, ACC and medial prefrontal cortex (MPFC), while no increased regional ALFF was observed.

**Conclusion:**

TIPS insertion alters cirrhotic patients' ALFF patterns in the resting state, which may imply different short-term and moderate-term effects on cirrhotic patients, i.e., both impairment and compensatory mechanism of brain functions in peri-TIPS and continuous impairment of brain function 3 months following TIPS.

## Introduction

Transjugular intrahepatic portosystemic shunt (TIPS) is a percutaneously created shunt through the liver parenchyma connecting the right or left portal vein to one of the three main hepatic veins [1], which is used increasingly in patients with variceal hemorrhage and refractory ascites. Although TIPS insertion is more effective than other alternative treatments such as endoscopic sclerotherapy in prevention of recurrent variceal bleeding [2], [3], the majority of controlled trials [2], [3], [4] and two meta-analyses [5], [6] demonstrated that it also increased the risk of portosystemic hepatic encephalopathy (HE) and had no survival benefit. Therefore, some authors considered TIPS as a double-edged sword and did not recommend it as a first-line treatment for the prevention of variceal rebleeding [7].

HE is one main complication of TIPS, with prevalence of approximate 30% in patients who underwent this insertion therapy. Many authors reported that the majority of post-TIPS HE happened within the first three months [8], [9]. Kramer et al. [10] first demonstrated TIPS can aggravate the cognitive impairment in patients without HE in a 6-month follow up observation using several neurophysiological tests e.g., event-related (P300) evoked potentials. However, the exact neuropathophysiological mechanism of TIPS's effects on brain function, especially within the three months following TIPS, has not been fully elucidated.

**Table 1:** Demographics and clinical data of pre-TIPS cirrhotic patients and healthy controls.

Protocols	HC (n = 16)	Patients (n = 16)	*P* value
Sex (M/F)	9/7	9/7	1^a^
Age (±SD), y	47.50±8.97	51.00±8.76	0.26^b^
Venous blood ammonia (μmol/L)		35.13±23.19	
bilirubin (μmol/L )		25.14±9.14	
Albumin (g/L)		36.11±3.81	
prothrombin time value (quick)		14.36±1.89	
Child-Pugh scale			
A		12	
B		4	
C		0	
NCT-A (s)	44.41±9.14	52.93±12.38	0.01^b^
DST (score)	45.00±11.32	34.43±9.53	<0.01^b^
Before TIPS		16	
No HE		13	
HE or MHE		3MHE	
Median 8 days after TIPS		10	
No HE		8	
HE or MHE		2 MHE	
Median 3 months after TIPS		7	
No HE		5	
HE or MHE		1HE#,1MHE	

Values are expressed as mean ± SD. TIPS  =  Transjugular intrahepatic portosystemic shunt; HC  =  healthy control; M  =  male; F  =  female; SD  =  standard deviation; NCT  =  number connecting-A; DST  =  digit symbol test; HE  =  hepatic encephalopathy; MHE  =  minimal hepatic encephalopathy.

a The *P* value for gender distribution in the two groups was obtained by chi-square test.

b The *P* value for age and neuropsychological tests difference between the two groups was obtained by two sample *t* test.

#The other HE patient died thus unavailable for follow-up MRI studies.

Functional neuroimaging plays an important role in uncovering functional abnormality of the brain in many brain diseases. A very recent study by Iversen *et al*. [11] first used positron emission tomography (PET) to measure the cerebral blood flow (CBF) both before and after the insertion of TIPS in cirrhosis patients, but no regional CBF changes were found following TIPS.

**Figure 1:**
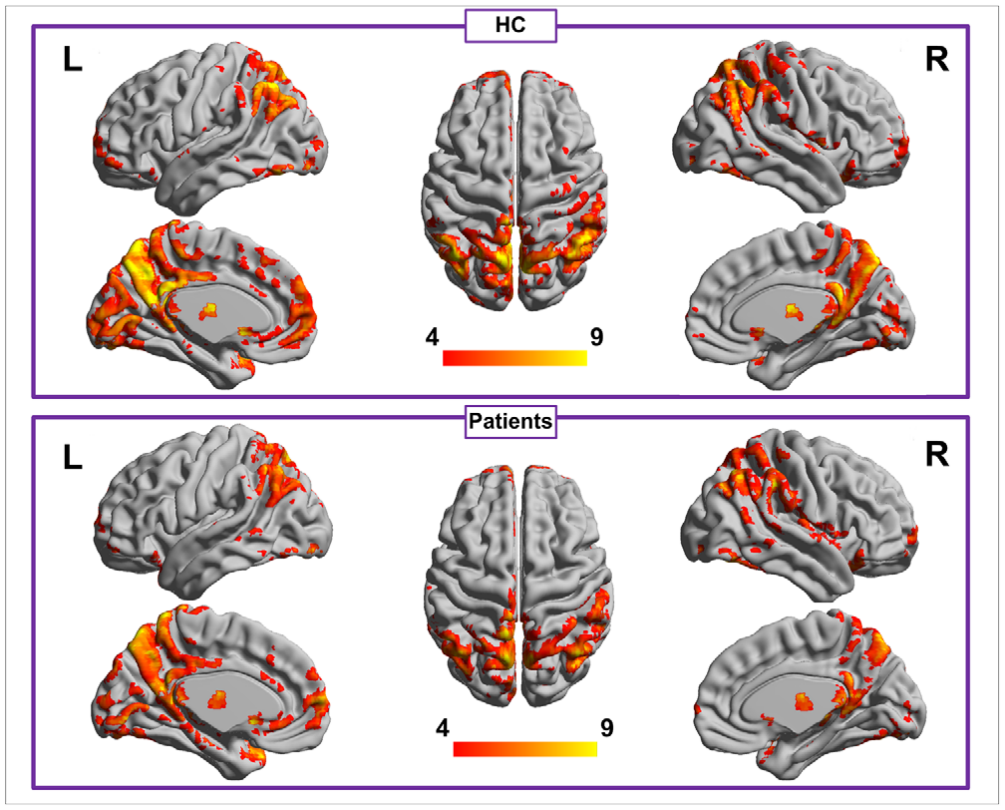
Within-group ALFF maps for the healthy control and cirrhotic patient groups at a corrected *P*<0.05. Within each group, PCC/PCu, MPFC, IPL, and occipital areas show high ALFF values. ALFF  =  amplitude of low frequency fluctuation; PCC  =  posterior cingulate cortex; PCu  =  precuneus; MPFC  =  medial prefrontal cortex; IPL  =  inferior parietal lobe.

Recently, resting-state functional MRI (rs-fMRI) has been developed as a new branch of the functional neuroimaging field. Increasing evidences have indicated that the pathophysiology of many neuro-cognitive dysfunctions including HE may be associated with the changes of spontaneous brain activity fluctuations measured during rs-fMRI [12], [13], [14]. When compared with other neuroimaging techniques, rs-fMRI has the advantages of no radiation exposure and high spatial resolution (compared to PET) and easy application (compared to task-driven fMRI) [15]. Some investigators have showed the clinical application of rs-fMRI in the presurgical localization of the epileptic source in individual patients with epilepsy [16], and monitoring the effect of different treatment on the brain function [17], [18]. Amplitude of low frequency fluctuation (ALFF) [19], one of the rs-fMRI analysis algorithms, has also been used as an effective fMRI algorithm in detecting the progression of diseases such as the Alzheimer's disease [20], as well as the HE [21]. Two recent fMRI studies with ALFF have demonstrated that the cirrhotic patients had widespreadly abnormal spontaneous brain activity, and the ALFF analysis algorithm could characterize the progression of the HE [21], [22].

**Figure 2:**
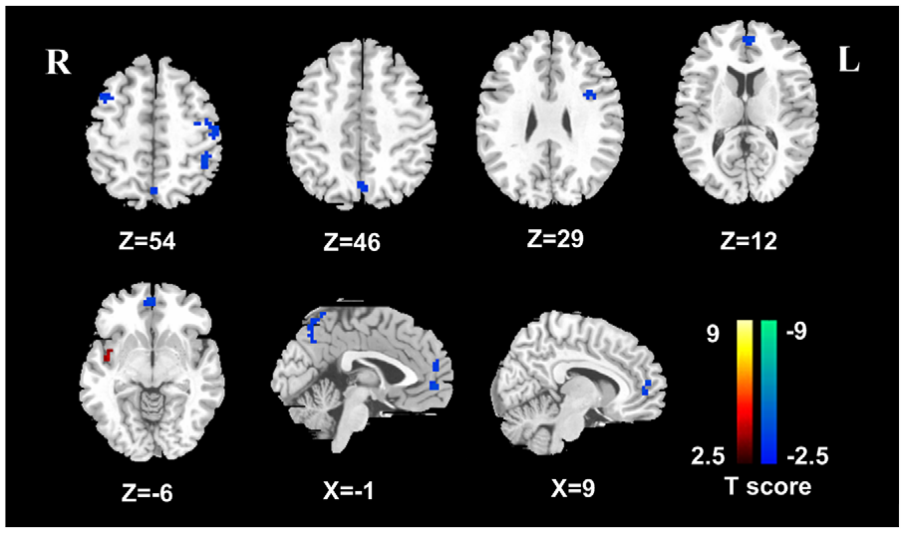
ALFF differences between cirrhotic patients before TIPS and healthy controls at a corrected *P*<0.05. Compared with the healthy controls, cirrhotic patients before TIPS showed widespread decreased ALFF in the frontal regions, involving left MPFC, ACC, SMA, bilateral DLPFC, right superior frontal cortex; and the parietal regions, such as the left PCu and angular cortex, and increased ALFF in the right insular cortex. (*P*<0.05, corrected for multiple comparisons). TIPS  =  Transjugular intrahepatic portosystemic shunt; ALFF  =  amplitude of low frequency fluctuation; MPFC  =  medial prefrontal cortex; ACC  =  anterior cingulate cortex; SMA  =  supplementary motor area; DLPFC  =  dorsal lateral prefrontal cortex; PCu  =  precuneus.

Based on the findings of previous behavioral studies that TIPS aggravated cognitive impairment in cirrhotic patients [10] and the high sensitivity of rs-fMRI in detecting the brain function changes, e.g., in the early phase of Alzheimer's disease [15], [23], we hypothesize that dynamic brain function changes in cirrhotic patients who underwent TIPS might be observed by rs-fMRI. To the best of our knowledge, no fMRI studies have been performed in the TIPS's effect on brain function. The analysis of dynamic intrinsic brain activity changes by using rs-fMRI in patients underwent TIPS may have great potential to improve the understanding of the pathophysiology of TIPS's effects on brain function, aid monitoring the treatment of TIPS and prewarning the postsurgical HE. To test our hypothesis, we adopt an unbiased whole-brain rs-fMRI analysis algorithm-ALFF to investigate the dynamics of brain function changes in cirrhotic patients after TIPS insertion.

**Figure 3:**
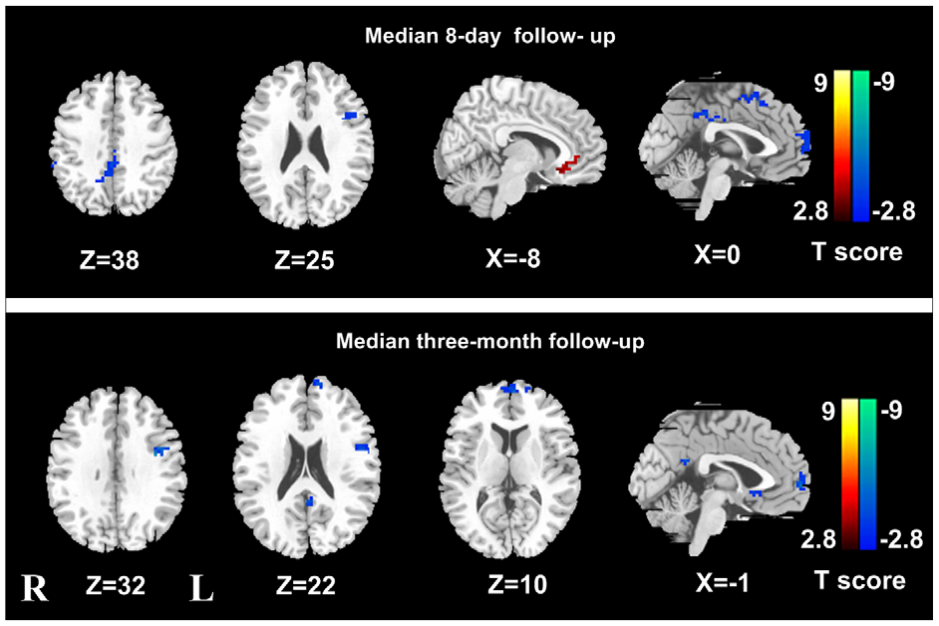
ALFF differences between patients in pre-TIPS and post-TIPS examinations. In the median 8-day follow-up examinations, post-TIPS patients demonstrated decreased ALFF in the left middle/posterior cingulate cortex, right superior frontal cortex, left DLPFC, SMA and increased ALFF in the left ACC and MPFC. In three months following TIPS, patients with TIPS insertion showed decreased ALFF in the left PCC, left DLPFC, left ACC and left MPFC, but no increased regional ALFF was detected. TIPS  =  Transjugular intrahepatic portosystemic shunt; ALFF  =  amplitude of low frequency fluctuation; DLPFC  =  dorsal lateral prefrontal cortex; SMA  =  supplementary motor area; ACC  =  anterior cingulate cortex; MPFC  =  medial prefrontal cortex; PCC  =  posterior cingulate cortex.

## Materials and Methods

### Subjects

This study was approved by the Medical Research Ethics Committee of Jinling Hospital and Clinical School of Medical College at Nanjing University. Written informed consents were obtained from all the participants before the study. Sixteen patients (9 male, 7 female; mean age, 51.0±8.8 years) with cirrhosis and portal hypertension scheduled for TIPS in our research institute of general surgery were included in this prospective study. The inclusion criteria for recruitment of the patients were as follows: The patients had liver cirrhosis diagnosed on the basis of clinical and imaging features, the cirrhotic patients with recurrent esophageal varices (which cannot be controlled by internal medical and endoscopy) or ascites formation, without overt HE or any history of overt HE, without any MRI contraindication, no focal abnormality in routine structural MRI examinations, and aged 18 years or older. In this study, 11 of these 16 included patients had variceal rebleeding despite internal medical treatment with vasoconstrictive drugs and endoscopic sclerotherapy or ligation, 5 had ascites refractory to high-dose combination diuretics. Covered stent grafts (Fluency stent grafts: 8 mm×6 cm, manufactured by Angiomed GmbHCo. subsidiary of C.R. Bard, Inc. Germany) were used in this study, which were inserted according to standard methods and without complications [24]. Correct stent function was ascertained by the immediate fall in the portosystemic venous pressure gradient and by Doppler ultrasonography. The stent grafts used for TIPS in this study are constructed of nitinol, which has been proved to create markedly reduced susceptibility artifact compared with stainless steel stents and be safe for patients [25], [26]. Some published papers have performed MRI for visualization of these stent grafts after TIPS [25], [26]. Exclusion criteria for all the subjects were any drug abuse history; or translation more than 1.0 mm or rotation more than 1.0° during MR scanning. Demographics and clinical data for all the subjects were summarized in Table 1.

**Figure 4:**
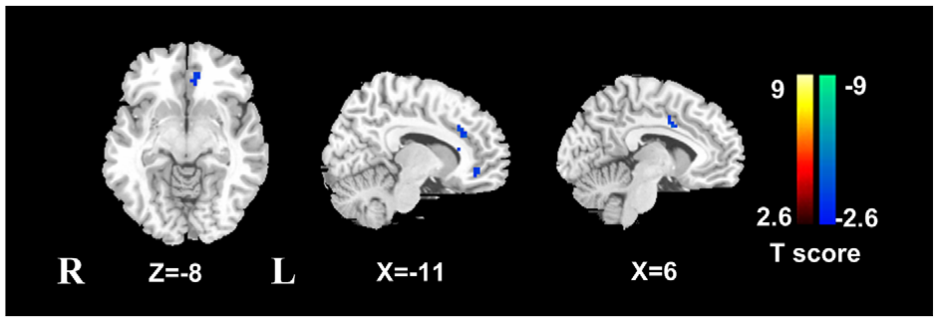
ALFF differences between post-TIPS cirrhotic patients in median 8-day and 3-month follow-up examinations. When compared with all patients in the median 8-day follow-up, patients in the 3-month follow-up examinations showed decrease ALFF in the left ACC/MPFC and right middle cingulate cortex. ALFF  =  amplitude of low frequency fluctuation; TIPS  =  Transjugular intrahepatic portosystemic shunt; ACC  =  anterior cingulate cortex; MPFC  =  medial prefrontal cortex.

All patients underwent neuropsychological tests including number connecting-A (NCT-A) and digit symbol test (DST). Minimal hepatic encephalopathy was diagnosed if the scores of at least one of the above-mentioned tests were beyond 2 standard deviation (SD) of the mean value for the age-matched healthy controls [27]. In this study, the control values from healthy controls were 44.41±9.14 (SD) sec for NCT-A score and 45.00±11.32 (SD) for DST score, and hence, cirrhotic patients with a NCT-A score higher than 63 sec or a DST score 22 or lower were regarded as MHE., Here, 16 healthy controls (mean age, 47.50±8.97 years) were included because of age- and gender-matching consideration. In fact, in one of our previously published studies, when 25 healthy controls with a similar age variance (mean age, 55±9 years) were included, the control values from healthy controls in that study were 46.32±9.09 (SD) sec for NCT-A score and 44.68±8.28 (SD) for DST score [28]. If we used the control value in that study, the same number of MHE patients would be defined in the present study.

**Table 2:** Regions showing ALFF differences between the pre-TIPS cirrhotic patients and healthy controls.

Brain regions	BA	MNI Coordinates (mm)	ΔVol (mm^3^)	Peak *t* value
		(x, y, z)		
Left PCu	5/7	−3,−63,51	972	−2.51
Right parietal lobe (angular cortex)	40	3, −39,66	756	−2.55
Left MPFC	10	0,51,15	864	−2.61
Right ACC	32/9	9,36,21	459	−2.55
Right SMA	6	3,0,60	567	−2.52
Left DLPFC	9/46	−42,6,24	648	−2.47
Right DLPFC	9/46	45,24,27	513	−2.54
Right superior frontal cortex	8	18,36,42	891	−2.46
Right insular cortex	13/22	42,−6,−9	1080	+5.00

Positive sign in the peak *t*-score represents increase, and negative sign represents decrease. All *P* <0.05, corrected for multiple comparisons using AlphaSim program. ΔVol: volume difference; ALFF  =  amplitude of low frequency fluctuation; TIPS  =  Transjugular intrahepatic portosystemic shunt; BA  =  Brodmann's area; PCu  =  precuneus; MPFC  =  medial prefrontal cortex; ACC  =  anterior cingulate cortex; SMA  =  supplementary motor area; DLPFC  =  dorsal lateral prefrontal cortex.

Laboratory parameters including prothrombin time, protein metabolism test, and venous blood ammonia results were obtained from all patients within one week before MR scanning to assess the severity of liver disease. The grade of hepatic function was determined according to the Child-Pugh score [29].

Sixteen age-and sex-matched healthy controls (Table 1) were recruited from the local community by advertisements. All healthy controls had no diseases of the liver (cirrhosis, hepatitis, liver tumors or extrahepatic portal vein obstruction) and of other systems, with no abnormal findings in abdominal ultrasound scans (performed within one week before MR scan) and conventional brain MR imaging. Furthermore, all the subjects in our study were free of any current signs and history of psychiatric illness (interviewed by the psychiatrist in our hospital), cardiovascular diseases, or other known diseases that would affect brain functions. Healthy controls underwent neuropsychological tests before the MR scanning. No laboratory tests were performed thus unavailable for them.

**Table 3:** Regions showing ALFF differences of the patients after TIPS in paired comparison.

Time	Brain regions	BA	MNI coordinates (mm)	ΔVol (mm^3^)	Peak *t* value
			(x, y, z)		
Median 8-day follow-up	Left middle/posterior cingulate cortex	31/23	−3,−39,39	486	−2.98
	Right SMA	6	3,18,51	459	−3.08
	Left DLPFC	9/45	−54,9,21	459	−3.12
	Left MPFC	10	−3,63,3	594	−3.01
	Left ACC	10/32	−12,30,−12	594	+4.33
Median 3-month follow-up	Left DLPFC	9/6	−51,0,27	648	−3.24
	Left ACC	25	−9,12,−6	540	−3,13
	Left MPFC	10	−6,66,0	756	−3.45
	Left PCC	23	−6,−45,21	324	−3.28^*^

Positive sign in the peak *t*-score represents increase, and negative sign represents decrease. All *P*<0.05, corrected for multiple comparisons using AlphaSim program. ^*^The region in the posterior cingulate cortex survived the height but not the extent threshold. ΔVol: volume difference; ALFF  =  amplitude of low frequency fluctuation; TIPS  =  Transjugular intrahepatic portosystemic shunt; BA  =  Brodmann's area; SMA  =  supplementary motor area; DLPFC  =  dorsal lateral prefrontal cortex; MPFC  =  medial prefrontal cortex; ACC  =  anterior cingulate cortex; PCC  =  posterior cingulate cortex.

**Figure 5:**
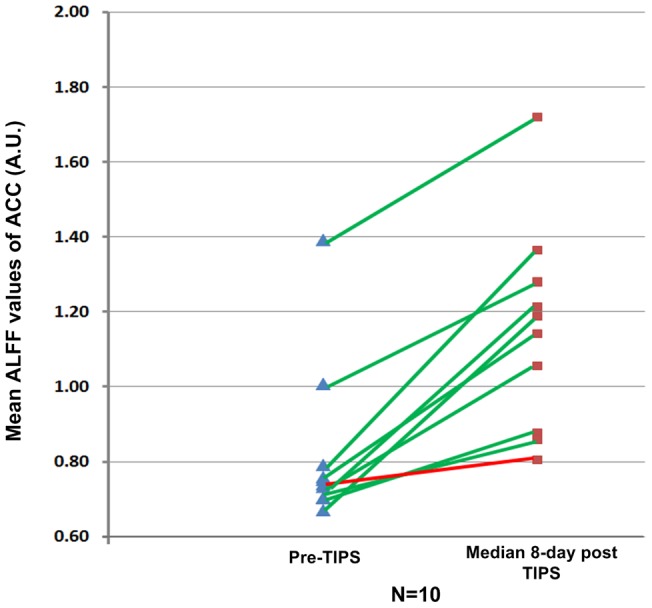
ALFF values of ACC in ten cirrhotic patients who underwent per-TIPS and median 8-day follow-up MRI studies. Of these ten patients undergoing the 8-day follow-up MRI studies, only one with slight increase rate of ALFF values in the ACC (<10%, red color line) experienced overt HE during the 3-month follow-up observation. All other nine patients with moderate to large increase rate of ACC ALFF values (>20%) did not have overt HE in the following follow-up observations (green color line). Blue triangle indicates ACC ALFF values in ten patients before TIPS; Red rectangle represents ACC ALFF values in the same patients who repeated the 8-day follow-up MRI studies. ALFF  =  amplitude of low frequency fluctuation; ACC  =  anterior cingulate cortex; TIPS  =  Transjugular intrahepatic portosystemic shunt.

### MRI data acquisition

All the subjects were examined with MR imaging. Ten post-TIPS patients studied again in a median 8 days (range, 6–13 days) follow-up after TIPS, 7 patients also participated in a median 3-month (range, 3–8 months) follow-up examinations (5 of them were the same as those in the 8-day follow-up MRI study). MRI data were acquired on a 3 Tesla MR scanner (TIM Trio, Siemens Medical Solutions, Erlangen, Germany). The participants were instructed to lie quietly and keep their eyes closed but be awake in the MR scanner. Foam pad was used to minimize the head motion of all subjects. Axial anatomical images were acquired using a T1- FLASH sequence (TR/TE  = 350 ms/2.46 ms, matrix  = 320×256, field of view (FOV)  = 240×240mm^2^, slice thickness/gap  = 4.0 mm/0.4 mm). Thirty slices aligned along the anterior commissure-post commissure line were acquired. Functional images were subsequently obtained at the same slice orientation and thickness as the anatomical images with a single-shot, gradient-recalled echo planar imaging sequence (TR/TE  = 2000 ms/30 ms, FOV  = 240×240mm^2^, flip angle  = 90°, matrix  = 64×64, voxel size  = 3.75×3.75×4mm^3^). A total of 250 brain volumes were collected, resulting in a total scan time of 500 s.

### Data preprocessing

Functional imaging data were pre-processed using SPM8 software package (http://www.fil.ion.ucl.ac.uk/spm). The first 10 volumes of functional images were excluded for magnetization to reach steady state and allowing adaptation of the subjects to the scanning noise. The remaining 240 consecutive volumes were used for data analysis. Slice-timing adjustment and realignment for head-motion correction were performed. No translation or rotation parameters in any given data set exceeded 1mm or 1.0°, and no group differences were found in respect to head translation and rotation (both *P>*0.05). The functional images were then spatially normalized to stereotaxic coordinates of the standard Montreal Neurological Institute (MNI) and resampled into voxel size of 3×3×3 mm^3^, and then smoothed by convolution with an isotropic Gaussian kernel (FWHW  = 4 mm). After smoothing, subsequent data preprocessing included removal of linear trends and temporally filtering (band pass, 0.01–0.08 Hz) to remove the effects of low-frequency drift and high-frequency noises.

### ALFF analysis

We applied REST software (http://resting-fmri.sourceforge.net) to calculate the ALFF. The calculation procedure has been described in the previous studies [19], [30]. Briefly, the time course was first converted to the frequency domain using Fast Fourier Transform. The square root of the power spectrum was computed and then averaged across 0.01–0.08 Hz at each voxel. This averaged square root was taken as the ALFF [19]. For standardization purpose, the ALFF of each voxel was divided by the global mean ALFF value. The standardized ALFF value in a given voxel reflects the degree of its raw ALFF value relative to the average ALFF value of the whole brain.

### Statistical analysis

#### Within-group ALFF analysis

To explore the within-group whole brain ALFF patterns, a random-effect one-sample *t* tests were performed on the individual ALFF maps in a voxel-wise way for patient group before TIPS and healthy control group. Significant thresholds were set at a corrected *P*<0.05, with multiple comparison correction using false discovery rate (FDR) criterion. The group-level ALFF maps were then visualized with the BrainNet Viewer (http://www.nitrc.org/projects/bnv/).

#### Between-groups ALFF analysis

To compare the spontaneous brain activity between cirrhotic patients scheduled for TIPS and healthy controls, a random-effects analysis two-sample *t* tests were calculated, with age, sex and head motion importing as covariates. To take into account of multiple comparisons, the result was corrected using AlphaSim program (http://afni.nih.gov/afni/docpdf/AlphaSim.pdf) determined by Monte Carlo simulation [31], thresholds were set at a corrected *P*<0.05 (Parameters were: single voxel *P* value  = 0.01, a minimum cluster size of 459 mm^3^, FWHM  = 4 mm, within a gray matter mask corresponding to the AAL atlas). To investigate how TIPS affect on patients' brain function, we used random-effects paired *t* tests to examine for any differences in ALFF values before and after TIPS (two follow-up MR examinations with two paired *t* tests respectively). In addition, we also used random-effects two-sample *t* tests to observe the difference of brain activity between the patients in median 8-day and 3-month follow-up MR examinations. The significant statistical threshold was likewise conducted using the aforementioned AlphaSim program.

Furthermore, to evaluate the potential of short term rs-fMRI to predict overt HE following TIPS, we selected the region showed most significant ALFF changes in median 8-day follow-up MR study as seed region and extracted its quantitive ALFF values. The regional ALFF change rate in the short term rs-fMRI was calculated using the following formula: ALFF change rate  =  (regional ALFF value_post-TIPS_ – regional ALFF value_pre-TIPS_)/regional ALFF value_pre-TIPS_, then compared between those who had and did not have episode of HE in the long term follow up.

## Results

### Clinical Characteristics

In the current study, compared with 16 healthy controls, 16 pre-TIPS patients showed no difference in age and gender, but worse neuropsychological performance (Table 1).

Three patients did not perform the follow up studies because of withdraw from the study. Ten of the remaining 13 patients were studied in a median 8-day (range, 6–13 days) after TIPS, and 7 patients underwent the median 3-month (range, 3–8 months) post-TIPS examinations (5 of these 7 patients also had participated in the median 8-day post-TIPS examinations). During the median 8-day follow-up observation, all 10 patients had improved symptoms, and no one had episodes of overt HE, while during the median 3-month period, 2 of the 3 pre-TIPS MHE patients had episodes of HE, and one HE patient died thus unavailable for follow-up MRI studies (this patient also had no MR data in the median 8-day follow-up), so only one HE patient performed the median 3-month MRI scanning. Other post-TIPS patients had no episode of HE in the up to 2 years follow-up observations assessed by phone interviews. Not all patients completed testing procedures (due to non-responding/refusals/death) at the above-mentioned follow-up duration.

The average blood ammonia levels were significantly different among the pre- and post-TIPS patients in each period, showing an increasing trend of median 8-day follow-up (30.30 ±24.28 µmol/L) < pre-TIPS (35.13±23.19 µmol/L) < median 3-month follow-up (65.02±35.15 µmol/L) (one-way analysis of variance, *F* = 3.75, *P* = 0.04), with significant difference between median 3-month follow-up and other groups (post-hoc test), but without significant difference between 8-day follow-up and pre-TIPS (*P* = 0.65). No other relevant changes of neuropsychological tests scores, biochemical, and hematological parameters were observed during the follow-up observations (all *P>*0.05).

### Within-group ALFF maps

Within each group, the posterior cingulate cortex (PCC)/precuneus (PCu) had a significant higher standardized ALFF value than the globe mean ALFF value. Other brain regions, including the medial prefrontal cortex (MPFC), inferior parietal lobe (IPL), and occipital areas also had high ALFF values (Figure 1).

### Group differences in ALFF

Compared with the healthy controls, cirrhotic patients before TIPS showed widespread decreased ALFF in the frontal region, involving left MPFC, right anterior cingulate cortex (ACC), supplementary motor area (SMA), bilateral dorsal lateral prefrontal cortex (DLPFC), right superior frontal cortex; and the parietal regions, such as left PCu and angular cortex, and increased ALFF in the right insular cortex (Figure 2, Table 2) (*P*<0.05, corrected for multiple comparisons). In the median 8-day follow-up examinations, compared with pre-TIPS ones, 10 post-TIPS patients demonstrated decreased ALFF in the left middle/posterior cingulate cortex, right superior frontal cortex, left DLPFC, SMA and increased ALFF in left ACC (Figure 3, Table 3). In 3 months following TIPS, cirrhotic patients with TIPS insertion showed decreased ALFF in the left PCC, left DLPFC, left ACC and left MPFC, but no increased regional ALFF was shown (Figure 3, Table 3). When all 7 patients in median 3-month follow up examination were compared with 10 patients in the median 8-day, only decreased ALFF was observed in the left ACC/MPFC (voxels: 702 mm^3^, peak *T* value: 3.41) and right middle cingulate cortex (voxels: 540 mm^3^, peak *T* value: 2.76) in patients in 3-month follow up observation (Figure 4).

In the short term (median 8-day follow-up) rs-fMRI study, ACC showed most significantly changed ALFF values (with maximum peak *T* value, see Table 3), then it was extracted as a mask for region of interest (ROI), then this mask was applied to all the patients and its mean ALFF values were calculated using the REST software [32]. We compared the mean ALFF values of the ACC of pre-TIPS, 8-day and 3-month follow-up examinations, and found there was a trend of decreased mean ALFF values of median 8-day follow-up (1.15±0.27) > pre-TIPS (0.82±0.22) > median 3-month follow-up (0.77±0.13) (one-way analysis of variance, *F* = 7.76, *P*<0.01), with significant differences of mean ALFF values between 8-day follow-up and the other examinations. Furthermore, of these10 patients undergoing the short term MRI studies, only one patient had minimum increase rate of ALFF value in the ACC (increased by 7.31%), and he experienced three episodes of overt HE during the following 3-month follow-up duration (Figure 5). All other nine patients had a moderate to lager increase rate of ALFF value in the ACC (range: 22.46%–107.22%, mean: 49.19%), and they did not report any episodes of overt HE in the up to 2 years follow-up. During the 3-month follow-up observation, two patients had HE and one died.

We further preformed the correlation analysis between the mean ACC ALFF values changes and the neurological tests scores, as well as changes of neurological tests scores in each follow-up after TIPS, and found that the ALFF changes in median 8-day follow-up (median 8-day follow-up – before TIPS) negatively correlated with the NCT scores in this peri-TIPS time point (*r* = −0.68, *P* = 0.03), suggesting that the patients with less increased ALFF values of the ACC shortly after TIPS would have worse NCT results. No other significant correlation between ALFF and neurological tests scores was found.

## Discussion

This study found different short-term and moderate-term effects of TIPS insertion on cirrhotic patients, i.e., both impairment and compensatory mechanism of brain function in peri-TIPS and continuous impairment of brain function in 3 months following TIPS.

Amplitude of low frequency fluctuation (ALFF) developed by Zang *et al*. [19] is a new index for brain functional analysis, which has been widely used in studies of various mental disorders e.g., schizophrenia [33], attention deficit hyperactivity disorder [19], and Alzheimer's disease [20]. Although the exact biological mechanism of ALFF remains unclear to date, many scholars consider that these spontaneous low-frequency fluctuation changes in blood oxygenation level dependent signal are associated with local neuronal activity [30], [34]. It should be noted that different fMRI analysis algorithms may reflect different aspects of comprehensive brain functions. Compared with other algorithms such as functional connectivity analysis, ALFF has the advantage of directly reflecting the amplitude or intensity of spontaneous activity, as shown in the present study and several other studies [30], [35].

In this study, pre-TIPS patients already had widespread aberrant spontaneous brain activities. The results were quite consistent with a recent fMRI study of HE with ALFF analysis [21]. It should be noted that many regions with decreased ALFF were located in the default mode network (DMN), including MPFC, ACC and PCu, which is consistent with a recent rs-fMRI study with ALFF in HE patients [21]. DMN [36] is thought to engage in the maintenance of the baseline brain activities, which are related to cognitions of self-awareness, episodic memory and interactive modulation between the internal mind activities and external tasks. The DMN abnormalities in patients with or without HE have been reported in a few task-driven [37] and rs-fMRI studies [12], [13], [38]. The present findings of decreased spontaneous brain activity in DMN suggested that pre-TIPS cirrhotic patients already had abnormal brain function.

Following TIPS, 2 of the 3 pre-TIPS MHE patients in our study had episodes of HE in the median 3-month follow-up, this is consistent with one previous report that patients with HE before TIPS had increased risk of post-TIPS HE [8]. In addition, patients who underwent the median 8-day follow-up rs-fMRI showed bilaterally changed (either decreased or increased) regional ALFF values, while in the median 3-month follow up observation, patients displayed persistently decreased regional ALFF values. These findings suggested that TIPS insertion may have different short-term and moderate-term effects on cirrhotic patients. In particular, there might co-exist impairment and compensatory mechanisms of brain functions in peri-TIPS and continuous impairment of brain function in 3 months following TIPS. Two recent fMRI studies with ALFF analysis algorithm also reported both decreased and increased regional ALFF values in cirrhotic patients [21], [22]. However, in a prospective PET study, Iversen *et al*. [11] measured cerebral blood flow (CBF) by [^15^O]-water PET in 9 cirrhotic patients before and median 11-day post TIPS, but found no significant CBF changes. The discrepancy between ours and the above-mentioned PET study might be due to differences in imaging techniques. Resting-state fMRI using in this study has been used in detecting the slight brain function changes [15], e.g., in the early phase of Alzheimer's disease [15], [39] and HE [12].

ACC in the present study showed increased brain activity in the median 8-day follow-up while decreased brain activity in the median 3-month following TIPS. ACC is a center involved in a form of attention which severs to regular both cognitive and emotional processing [40]. Interestingly, attention defect is a fundamental aspect of HE [41], [42], and abnormal ACC function in cirrhotic patients had been reported by a previous PET study [43]. The dynamically changed ACC activity may be partially supported by the dynamic changes of blood ammonia in the present study, whose levels showed an increasing trend of median 8 day follow-up < pre-TIPS < median 3-month follow-up. Ammonia plays an important role in the pathogenesis of HE. The ammonia-related cellular alterations are considered to be important for the development of HE [44]. Additionally, of 10 patients undergoing the median 8-day follow-up, only one patient who showed minimum increase rate of the ACC activity (<10%) had some episodes of HE in the 3-month follow-up. All other nine patients with larger increase rate (>20%) did not experience HE. If these results are confirmed by further studies with more patients, they may imply that patients with slight increase rate of the ACC activity shortly after TIPS might have propensity to develop into overt HE, suggesting that rs-fMRI might have a potential to predict the development of postsurgical HE.

The current study has some limitations. First, as a preliminary study, our results are limited to a small patient cohort, and the follow-up examinations are only available for some of the patients after TIPS insertion, especially in the median 3-month follow-up the sample size is small, thus, the statistical power for the data analysis in this study is limited, and further studies with more patients in each follow up period are warranted to confirm these findings. Second, not all post-TIPS patients performed all the follow-up test procedures, and the results of longer follow-up MR examinations were not included in this study. Third, it is possible that brain function change may be a consequence of HE, and ideally we need to include a control group of cirrhotic patients who did not undergo TIPS. However, based on the fact that none of cirrhotic patients had overt HE pre-TIPS and during the short-term follow-up, and the brain function of patients with TIPS insertion was already changed compared to pre-TIPS studies, we believe that this altered brain function is primarily due to the TIPS procedure itself. Fourth, the exact biologic mechanism of ALFF we used here remains unclear to date, in addition, the dynamic changes of ALFF values and blood ammonia levels in patients after TIPS treatment in the present study need to be confirmed by further studies.

In conclusion, the present study showed that TIPS insertion altered patients' brain function, which might have different short-and moderate-term effects. Resting- state fMRI with ALFF analysis might potentially supply a novel way to understand the neuro-pathophysiological mechanism of TIPS's effects on brain function.
